# Changes in Mobility of the Golden Hamster with Induction of an IL-1-Induced Arthritis

**DOI:** 10.1155/S096293519400027X

**Published:** 1994

**Authors:** I. G. Otterness, M. L. Bliven, A. J. Milici

**Affiliations:** Department of Immunology and Infectious Diseases Central Research Division Pfizer Inc. Groton CT 06340 USA

## Abstract

Many studies in animals have examined biochemical, immune and histological changes during arthritis; however, the study of the effects of arthritis on mobility has been largely neglected. Interleukin-1, administered by the intraarticular route into hamster knee joints, resulted in inhibition of spontaneous wheel running activity; however, the effect was transient, lasting only through the evening following IL-1 administration. A further injection of IL-1 2 days later showed still greater inhibition of running. The effect again did not extend beyond the first evening after injection. IL-1α and IL-1β showed equivalent effects on mobility, and no evidence was seen for cooperative interaction between them. A 50% inhibition of running occurred at a dose of approximately 10 ng/knee of IL-1α. The effect appeared not to be systemic since intraperitoneal injection required microgram amounts of IL-1 for an equivalent inhibition. At the time mobility had been restored to normal, histological examination showed the continued presence of inflammatory cells, soft tissue swelling and cartilage proteoglycan loss. These results suggest a lack of correlation between inhibition of mobility and histopathological changes in cartilage and soft tissue.


Mediators of Inflammation 3, 199–204 (1994)

### Changes in mobility of the golden hamster with induction of an IL-1-induced arthritis

I. G. Otterness,[CA] M. L. Bliven and A. J. Milici

Department of Immunology and Infectious Diseases, Central Research Division, Pfizer Inc., Groton, CT 06340, USA

[CA] Corresponding Author

**MANY studies in animals have examined biochemical, immune and histological changes during arthritis; however, the study of the effects of arthritis on mobility has been largely neglected. Interleukin-1, administered by the intraarticular route into hamster knee joints, resulted in inhibition of spontaneous wheel running activity; however, the effect was transient, lasting only through the evening following IL-1 administration. A further injection of IL-1 2 days later showed still greater inhibition of running. The effect again did not extend beyond the first evening after injection. IL-1α and IL-1β showed equivalent effects on mobility, and no evidence was seen for cooperative interaction between them. A 50% inhibition of running occurred at a dose of approximately 10 ng/knee of IL-1α. The effect appeared not to be systemic since intraperitoneal injection required microgram amounts of IL-1 for an equivalent inhibition. At the time mobility had been restored to normal, histological examination showed the continued presence of inflammatory cells, soft tissue swelling and cartilage proteoglycan loss. These results suggest a lack of correlation between inhibition of mobility and histopathological changes in cartilage and soft tissue.**

**Key words:** Arthritis, Hamsters, IL-1, Mobility

### Introduction

Interleukin-1 (IL-1) has been suggested as an important mediator of arthritic disease. It has been shown *in vitro* to accelerate cartilage proteoglycan loss[1,2] and to inhibit proteoglycan synthesis.[3,4] IL-1 is known to be an inducer of other cytokines such as IL-6[5] and the colony stimulating factors (CSFs),[6] and induces adhesion molecules on endothelial cells[7] and chemotactic cytokines such as IL-8.[8] IL-1 can augment the immune response,[9,10] contribute to the formation of granulation tissue[11,12] and play a role in nonspecific immunity.[13] All these events are part of the inflammatory process.

IL-1 has been shown to produce inflammation and cartilage proteoglycan loss when injected intraarticularly in the rabbit,[14–16] mouse[17] and rat.[18] Moreover, IL-1 has been shown to have direct involvement in a murine[19] and in a rat model[20] of arthritis. Previously, it has been shown that systemic administration of IL-1 can lead to decreased locomotor activity in the rat, measured as rearing activity and movement from one quadrant of the cage to another.[21] The possibility was considered that a local arthritis might also affect locomotor activity. However, preliminary studies of activity in locomotor cages showed only minor effects of arthritis on movement, perhaps because of the limited range of movement possible in a 30 × 45 cm cage. A running wheel offered the possibility of extended running activity that could be modified by arthritis. However, the normal untrained rat shows highly variable running. In contrast, a hamster without training will travel many kilometres a night on a wheel or disk[22] and therefore seemed a better species in which to explore the effects of arthritis on mobility. Thus, the consequences of an arthritis induced by local intraarticular administration of IL-1 on mobility of the hamster were studied.

### Methods

*Animal care and housing:* Female Golden Syrian hamsters (*Mesocricetus auratus*) strain LAK.LVG(SYR) were purchased from Charles River Laboratories (Kingston, NY) at 100–110 g weight. They were maintained on a 10/14 light cycle with food and water *ad libitum* and were acclimatized to our facility for at least a week in standard housing before studies were initiated. Hamsters were placed into cages with 33 cm diameter wheels (Nalge, Rochester, NY) at least 3 days prior to manipulation.

*Intraarticular injections:* For intraarticular injection, hamsters were anaesthetized with sodium pentobarbital (80–100 mg/kg i.p.) generally 1–2 h before the dark cycle. The treatment groups were given identical intraarticular injections of 20 μl IL-1α or IL-1β, prepared and characterized as described

© 1994 Rapid Communications of Oxford Ltd previously,[23,24] or phosphate buffered saline (PBS) vehicle delivered with a 30-gauge needle into both knees or no injection. The hamsters were weighed, and returned to their cages. Wheel revolutions were continuously recorded. On the day of termination of the experiment, the animals were again anaesthetized, and the left knee was taken for histological analysis.

*Data collection:* Wheels were fitted with magnet reed switches. A 386/25 MHz computer was interfaced with a 24-channel I/O board to collect real-time data. Wheel turns were recorded automatically in 1 min bins and cumulative wheel turns per day recorded. The wheel turns per day for each animal were recorded for 3 to 5 days prior to intraarticular injection and were averaged to determine a normal daily distance (NDD) for each animal. Fractional daily distance for all other days was calculated by dividing the hamster's distance on that day by the prior NDD. Generally, daily data are presented as the average of the fractional distance of all hamsters in the experimental group ± S.D. For data in which the movement characteristics for individual animals were examined in detail, wheel revolutions were collected in 6 min (0.1 h) intervals. This facilitated analysis on a PC by reducing the analysis of data to 240 points per day.

*Histology:* The hind limbs of control and IL-1 treated animals were removed and fixed overnight in 10% neutral buffered formalin (4°C). Following fixation, the knees were decalcified for 72 h in a 1 : 1 mixture of 8 N formic acid and 1 N sodium formate (Kristensen's solution). The knees were then rinsed for 24 h with cold tap water, and routinely processed and embedded in paraplast plus. Great care was used in the mounting of the tissues to ensure that sectioning would begin perpendicular to the medial side of the knee. Sections 3 μm thick were cut from the medial to lateral side of the medial condyle. The sections were numbered and approximately matched sections from all animals were routinely stained with safranin O and fast green and examined using a light microscope.

### Results

*Normal running pattern:* The hamsters, when placed into a cage with a running wheel, quickly adapted to a consistent running pattern. They generally explore the cage, shortly discovering the wheel. Most are soon carrying out tentative exploration of the wheel motion, and within an hour, most are running as adeptly as seasoned runners. Almost independently of the time of day when they are placed in the cage, they will run greater than 90% of what will become their standard running distance that first night. Except for the exploratory behaviour of the first day, the daily running distance of the normal hamster appears largely constant from day to day over a 10–12 day experimental period, and occurs exclusively during the dark portion of the light cycle.

*Intraarticular IL-1α:* As an initial test to determine whether IL-1 induced inflammation would affect running, we chose to examine an amount of IL-1α (40 ng) that was four times the amount used to give inflammation, inhibition of proteoglycan synthesis and, after three injections, frank proteoglycan loss in the mouse.[17] The amount reflected a compromise between the differences in dose scaling based on surface area (3 ×) and body weight (5 ×) of a 125 g hamster and a 25 g mouse.[25] Results of a typical experiment are shown in Fig. 1. The normal nightly distance was determined using the 5 days of running prior to the IL-1 injection. After intraarticular IL-1 injection, there is approximately a 40% decrease from the normal daily running distance compared with saline injected controls.

The hamsters were allowed to run for a second night after the primary IL-1 treatment. Running distance was essentially normal. A second injection of IL-1 was then given. As shown in Fig. 1, a much more dramatic inhibition of running was observed after the second exposure to IL-1. In this experiment a mean decrease of 84% from the NDD was found. Surprisingly, even with this much more pronounced suppression of running on night 2, when left to run again on the following night, they ran essentially normal distances.

To examine the relationship between tissue inflammation, cartilage proteoglycan loss, and restoration of mobility, histological sections were taken of the IL-1-injected, the PBS injected, and the control animals after the animals had completed their second night's run, approximately 40 h following their second intraarticular IL-1 injection.

Histological sections from the PBS injected controls were free from all signs of inflammation. The

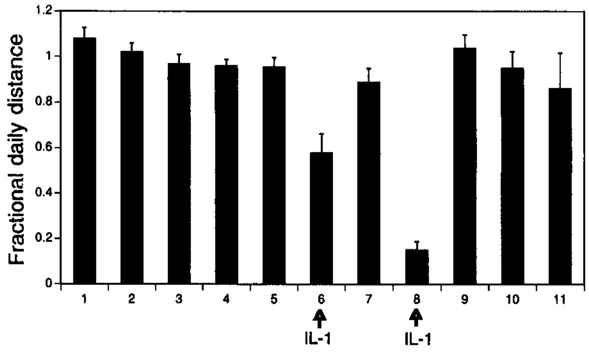

FIG. 1. The effect of intraarticular IL-1 on normal nightly distance. Hamsters ($n = 12$) were allowed to run for 5 nights and a normal nightly distance calculated (NDD = 9.3 ± 0.3 km). Six hamsters were injected with 40 ng IL-1α and six were injected with saline. The NDD was recorded for the next 2 nights. The hamsters were again injected with 40 ng of IL-1α or saline and allowed to run for another 2 nights.

soft tissue was free of leukocyte influx (Fig. 2A) and the cartilage proteoglycan staining was normal (Fig. 3A). Sections from the IL-1 treated animals showed clear and unmistakable inflammation of the surrounding soft tissue. There was a heavy leukocyte infiltration into both the deep and superficial soft tissue (Fig. 2B). There was an extensive loss of proteoglycan from the non-calcified layer of the cartilage as demonstrated by loss of safranin O staining, particularly in the pericellular regions which show the most intense staining (Fig. 3B). The synovial fluid continued to show evidence of a mild cellular infiltration and residual fibrin deposits. No comparable changes were observed in either the saline injected or control, non-injected hamsters.

*Dose response:* The effect of the amount of IL-1 on normal daily distance was examined. IL-1α was given intraarticularly in amounts of 10, 20 and 40 ng. Good inhibition of running by IL-1 was observed at all three doses with a dose responsiveness inhibition of nightly distance (Fig. 4). The $ED_{50}$ is estimated to be about 10 ng/joint. As was observed in the previous experiments, the inhibition of running was much more extensive after the second IL-1 injection when compared with the first injection of IL-1.

*Effect of IL-1α* vs. *IL-1β:* Experiments were carried out to determine whether there was a detectable difference in activity between IL-1α and IL-1β, and whether there was any evidence of a synergistic interaction between the two forms of IL-1. In previous experiments, 20 ng of IL-1 intraarticularly gave an intermediate response and therefore, that dose was chosen to be tested. One group received 20 ng of IL-1α, a second group received 20 ng IL-1β, a third

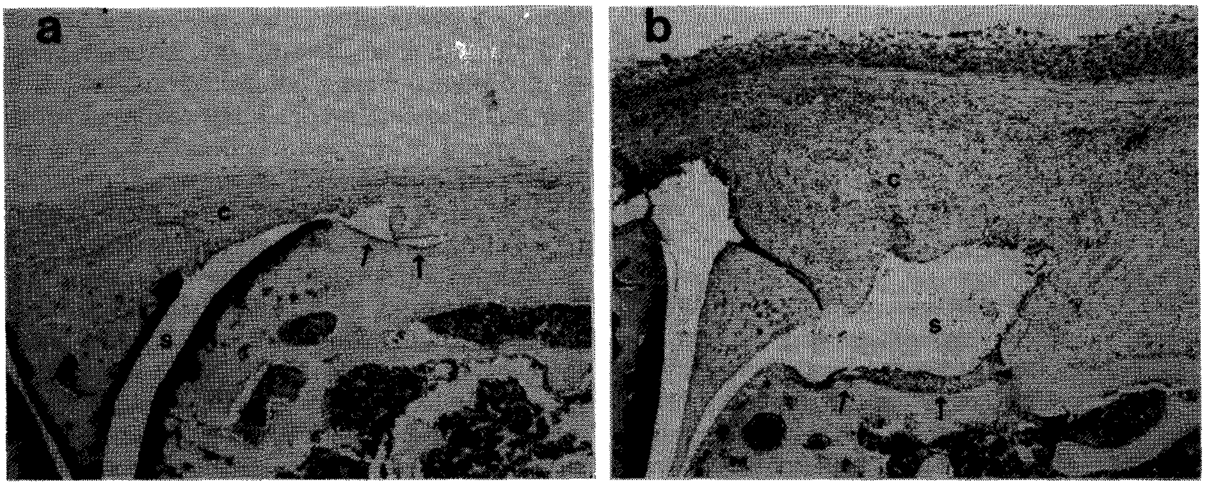

FIG. 2. Histology of the joint capsule 40 h after the second intraarticular injection saline (a), or 40 ng of IL-1α (b). The tissue sections were stained with safranin O and counterstained with fast green. In Fig. a, the typical appearance of a non-inflamed knee can be seen. There were few cells in the soft connective tissue surrounding the joint (c) and there was no hypertrophy of the synovial lining cells (arrows). In contrast, the effects of the IL-1a injections were clearly visible (Fig. b). There was a perceptible increase in the thickness and cellular content of the soft connective tissue surrounding the joint. In addition, hypertrophy of the synovial lining cells (arrows) was readily apparent. (Magnification = 40 ×).

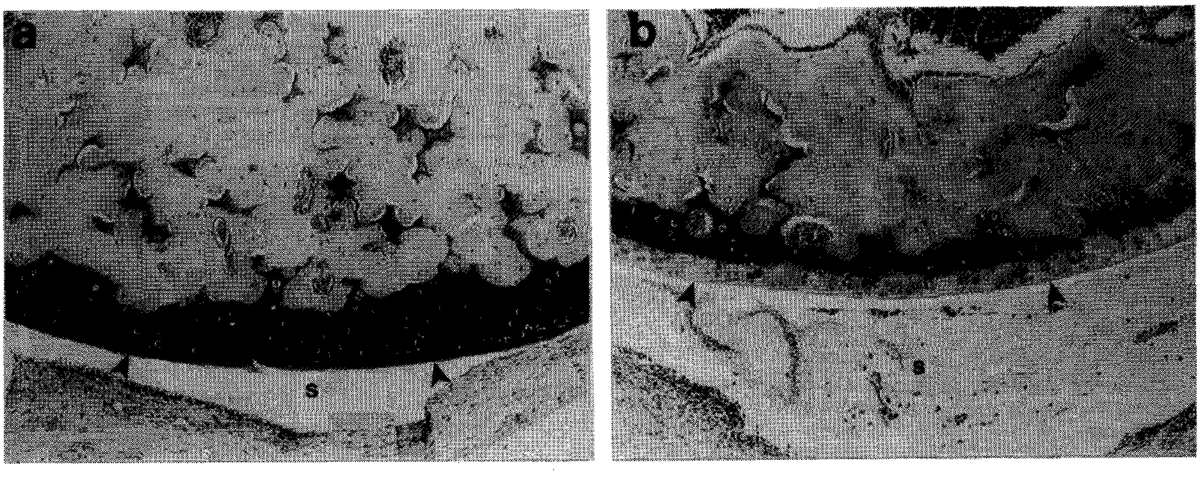

FIG. 3. Histology of the articular cartilage 40 h after the second intraarticular injection of saline (a), or of 40 ng of IL-1α (b). The saline injections did not result in any loss of proteoglycan from the articular surface of the femoral condyle (arrowheads) nor as any cell or proteinaceous material present in the synovial space, (s). In the IL-1α injected knees, the cartilage proteoglycan was depleted down to the tidemark (arrowheads) and there was an influx of cells and fluid into the joint spaces, (s). (Magnification = 100 ×).

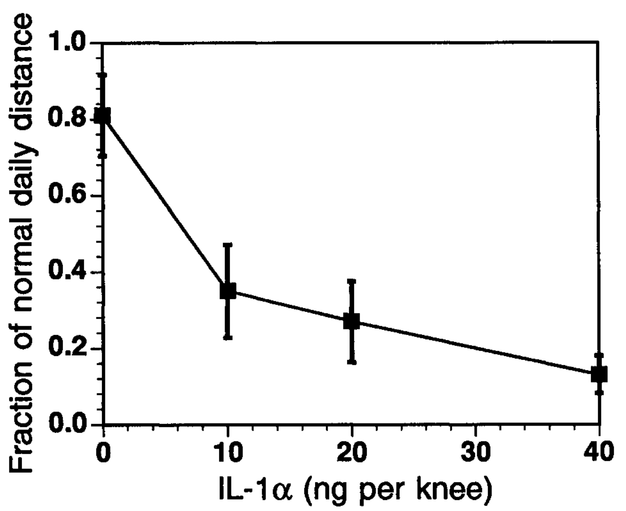

FIG. 4. Dose responsive inhibition of normal nightly distance after intraarticular injection of IL-1α. Hamsters were allowed to run for 3 nights and a normal nightly distance of 9.3 km/night was found. On the fourth day they were injected with IL-1α, and allowed to run for the following 2 nights. A second intraarticular injection of IL-1 was given and that night's distance recorded. The mean fractional distance for each group of six hamsters is plotted as a function of the intraarticular IL-1α.

group received 10 ng IL-1α and 10 ng of IL-1β, and a control group received only PBS. The results are given in Fig. 5. No difference was observed between the response to IL-1α and IL-1β, nor was there any evidence of either synergism or antagonism of one form of IL-1 for the other.

*Effect of intraperitoneal administration of IL-1α:* It has been shown previously that IL-1 given intraperitoneally can inhibit mobility.[21] Therefore the possibility was considered that the effect of intraarticularly administered IL-1 is either purely local, that is, caused by local changes at the site of injection, or might be caused by IL-1α or secondary mediators suffusing from the joint to cause a central inhibition of mobility. The effects of an 80 ng dose of IL-1α administered intraperitoneally (comparable with the highest intraarticular IL-1α dose) were compared with the effects of a 1 μg dose of IL-1α administered intraperitoneally, a dose that had previously been found necessary to inhibit mobility in the rat. The results (Fig. 6) demonstrate that no inhibition of mobility was observed after intraperitoneal administration of the 80 ng dose. However, a modest, but significant, inhibition of mobility was observed when 1 μg of IL-1α was administered intraperitoneally.

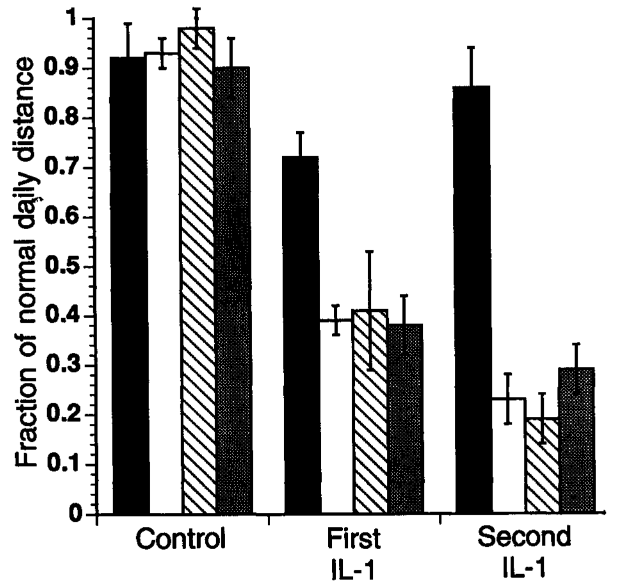

FIG. 5. Comparison of the effects of IL-1α and IL-1β on mobility inhibition in the hamster. Four groups of six hamsters were allowed to run for 4 nights and a normal nightly distance calculated for each hamster. The fractional nightly distance is shown for each group of hamsters for the night prior to the first IL-1 injection (night 1), for the night following the first IL-1 injection (night 0) and for the night following the second intraarticular injection (night 2). Four groups of six hamsters were examined. From left to right, the groups are 20 μl PBS, 20 ng IL-1α, 20 ng IL-1β, and 10 ng IL-1α plus 10 ng IL-1β.

### Discussion

Modelling changes that take place in mobility during human arthritis by studying the effects of arthritis in animals has not previously been done. Such studies in animals have advantages. Arthritis can be induced acutely and thus changes in mobility parameters can be referred directly back to previously normal values. The effects of different types of arthritis can be explored, and changes in mobility can be related to biochemical, histological and pain parameters studied during the experiment. As an initial test of this hypothesis, intraarticular IL-1 was used as an arthritogen principally because it causes a mild and reversible arthritis[26] and it was speculated that its effects would be primarily local under these circumstances. Thus, some of these conclusions may not be applicable to chronic arthritis where an overlay of systemic disease may change mobility in a fundamental way. Recognizing these qualifications, we still felt that simplicity dictated examination of the consequences of an acute local inflammation on mobility.

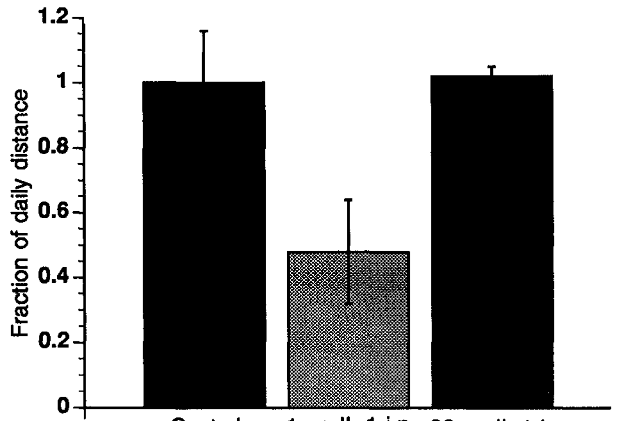

FIG. 6. Comparison of 80 ng IL-1α and 1 μg IL-1α given intraperitoneally. Groups of six hamsters were allowed to run for 3 nights, and a normal nightly distance computed for each hamster. On day 3, IL-1α was administered intraperitoneally and the nightly distance determined.

It was verified that intraarticular IL-1 induced joint inflammation in the hamster. Histologically, as expected from observations in other species, cell infiltration, soft tissue swelling, and proteoglycan loss were observed. The inflammation after a second IL-1 injection was greater than that after the first injection. A single intraarticular saline injection did not prime the joint to a greater response to a subsequent IL-1 injection (data not shown). It is suspected that the enhanced effect of the second IL-1 injection is due to its adminisration into an already inflamed joint. It has previously been shown that intraarticular IL-1 leads to potentiation of the smoldering arthritis induced with streptococcal cell wall peptidoglycan inducing development of much more extensive pannus, soft tissue swelling and cartilage destruction.[27] Thus, it appears that application of IL-1 to a site already infiltrated by inflammatory cells finds the tissue in a position to respond more readily to IL-1. The second intraarticular injection of IL-1 into a site is also characterized by a greater suppression of daily running distance than the first injection.

When the amount of IL-1 required to obtain an effect on mobility was measured, it was found that a good dose response was obtained in the range of 10–40 ng of IL-1 per joint. This is consistent with the amount of IL-1 which causes histopathological changes and cartilage depletion in mice.[17] Although this amount is well above the levels of IL-1 that can be measured in biological fluids, the rapid distribution and elimination in rodents of murine IL-1α and β[28] and human IL-1β[29,30] means that much larger amounts will be required by bolus injection to match that generated by continuous local production. Moreover, as described above, it is likely that the amount of IL-1 required to amplify an existing inflammatory lesion is much less than the amount of IL-1 required to elicit a full blown inflammation *de nouveau*. Thus, although it is considered that the amount of IL-1 utilized in these experiments is superphysiological, the changes produced probably reflect those seen following endogenous production.

IL-1α and IL-1β have both been shown to bind equally to the IL-I type I receptor whereas the type II receptor has been characterized by a preferential binding of IL-1β.[31] The comparable effectiveness of IL-1α and IL-1β in inhibition of mobility implies they are acting at the type I receptor. It has been shown previously by antibody blocking experiments that both IL-1α and IL-1β are involved in inflammation and inhibition of proteoglycan synthesis during arthritis.[19] However, Perretti *et al.*[32] have reported that peritoneal leukocyte infiltration is predominantly IL-1β dependent, and for effects more distal from the site of inflammation, i.e. weight loss[33] and fever,[34] a selective IL-1β dependency has been found.

The possibility that inhibition of mobility could be attributed to systemic rather than local effects was investigated. It has been found previously that continuous administration of IL-1α intraperitoneally in rats leads to inhibition of mobility without local joint pathology.[21] Thus we wondered if the amount of IL-1 given intraarticularly was sufficient to cause systemic inhibition of mobility. A dose of 1 μg of IL-1α injected intraperitoneally inhibited mobility with an effect that was comparable with 20 ng/joint intraarticularly (40 ng total). Sadaro and Dunn[35] using direct intracerebroventricular injection of 77 ng murine IL-1α into mice failed to observe a change in locomotor activity. However, they did observe a reduction in exploratory behaviour to novel stimuli. Dunn *et al.*[36] reported a decrease in exploratory behaviour at 10 ng i.p. in the rat, but no change in locomotor activity. Thus there is greater inhibition of mobility when IL-1 is given locally rather than systemically.

The nature of the joint inflammation may also affect the outcome of the mobility studies. By using IL-1, a mild inflammatory stimulus was chosen that leads to soft tissue swelling and proteoglycan loss. The articular cartilage is not innervated and it would not be expected to contribute to the loss of mobility. The synovium is, by contrast, known to be innervated and pressure sensitive efferents could transmit a signal suppressing mobility. Surprisingly, in spite of continuing inflammation and soft tissue swelling of the synovium, no impairment of mobility was found the second night after either the first or the second IL-1 injection. This may mean that IL-1 itself, or secondary mediators such as prostaglandins, directly sensitize pain receptors and thus there is impairment of mobility only while the mediators are present. Alternatively, the initial soft tissue swelling may lead to pain, but stress relaxation of the tissue occurs with time (i.e. accommodation of the tissue to distension), and, with stress relaxation, the hyperalgesic response may be lost in the absence of continuing additional inflammatory pressure. Finally, it is also possible that there has been direct evolutionary pressure toward preserving mobility. Hukkanen *et al.* and Mapp *et al.* have demonstrated the loss of sensory nerve fibres in inflamed synovium in adjuvant arthritic rats[37] and human rheumatoid arthritic patients,[38] respectively. Mobility compromised by joint inflammation would limit foraging and thus have a negative impact on survival and reproduction. Thus, the rapid restoration of mobility to normal in the presence of continuing inflammation of the joints may be an evolutionary outcome.

### References

1. Dingle JT, Saklatvala J, Hembry RM, Tyler JA, Fell HB, Jubb R. A cartilage catabolic factor. *Biochem J* 1979; **184**: 177–180.
2. Saklatvala JL, Pilsworth LMC, Sarsfield SJ, Gavrilovic J, Heath JK. Pig catabolin is a form of interleukin-1. *Biochem J* 1984; **224**: 461–466.
3. Tyler JE. Chondrocyte-mediated depletion of articular cartilage proteoglycans *in vitro*. *Biochem J* 1985; **225**: 493–507.

4. van den Berg WB, van de Loo FAJ, Zwarts WA, Otterness IG. Effects of murine recombinant interleukin 1 on intact homologous articular cartilage: a quantitative and autoradiographic study. *Ann Rheum Dis* 1988; **47**: 855–863.
5. McIntosh JDM, Mule JJ, Nordan RP, Rudikoff S, Lotze MT, Rosenberg SA. *In vivo* induction of IL-6 by administration of exogenous cytokines and detection of *de novo* serum levels of IL-6 in tumor-bearing mice. *J Immunol* 1989; **143**: 162-167.
6. Fibbe WE, Van Damme, AJB, Voogt PJ, Duinkerken N, Kluck PMC, Falkenburg JHF. Interleukin-1 (22-K factor) induces release of granulocyte-macrophage colony-stimulating activity from human mononuclear phagocytes. *Blood* 1986; **68**: 1316–1321.
7. Pohlman TH, Stanness KA, Beatty PG, Ochs DH, Harlan JM. An endothelial cell surface factor(s) induced *in vitro* by lipopolysaccharide, interleukin 1, tumor necrosis factor-α increases neutrophil adherence by a CDw 18-dependent mechanism. *J Immunol* 1986; **136**: 4548–4553.
8. DeMarco D, Kunkel SL. IL-1-induced gene expression of neutrophil activating peptide (IL-8) and monocyte chemotatic peptide in human synovial cells. *Biochem Biophys Res Commun* 1991; **174**: 411–416.
9. Kaye J, Gillis S, Mizel SB, *et al.* Growth of a cloned helper T cell line induced by a monoclonal antibody specific for the antigen receptor: interleukin 1 is required for the expression of receptors for interleukin-2. *J Immunol* 1984; **133**: 1339–1345.
10. Chiplunkar S, Langhorne J, Kaufmann SHE. Stimulation of B cell growth and differentiation by murine recombinant interleukin-1. *J Immunol* 1986; **137**: 3748–3752.
11. Dunn C, Hardee M, Staite ND. Acute and chronic inflammatory responses to local administration of recombinant IL-1α, IL-1β, TNFα and IFNγ in mice. *Agents Actions* 1989; **27**: 290–293.
12. Otterness IG, Golden HW, Brissette WH, Seymour PA, Daumy GO. Effects of continuously administered murine interleukin-1a: tolerance development and granuloma formation. *Infect Immun* 1989; **57**: 2742–2750.
13. Havell EA, Moldawer LL, Helfgott D, Kilian PL, Sehgal PB. Type I receptor blockade exacerbates murine listeriosis. *J Immunol* 1992; **148**: 1486–1492.
14. Pettipher ER, Higgs GA, Henderson B. Interleukin 1 induces leukocyte infiltration and cartilage proteoglycan degradation in the synovial joint. *Proc Natl Acad Sci USA* 1986; **83**: 8749–8753.
15. Dingle JT, Page TPD, King B, Bird DR. *In vivo* studies of articular tissue damage mediated by catabolin/interleukin 1. *Ann Rheum Dis* 1987; **46**: 527–533.
16. Feige U, Karbowski A, Rordorf-Adam C, Pataki A. Arthritis induced by continuous infusion of hr-interleukin-1a into the rabbit knee-joint. *Int J Tissue Reac* 1990; **11**: 225–238.
17. van de Loo AAJ, van den Berg WB. Effects of murine recombinant interleukin 1 on synovial joints in mice: measurement of patellar cartilage metabolism and joint inflammation. *Ann Rheum Dis* 1990; **49**: 238–245.
18. Chandraskar S, Harvey AK, Hurbey PS, Bendele AM. Arthritis induced by interleukin-1 is dependent on the site and frequency of intraarticular injection. *Clin Immunol Immunopathol* 1990; **55**: 382–400.
19. van de loo FAJ, Arntz OJ, Otterness IG, van den Berg WB. Protection against cartilage proteoglycan synthesis inhibition by antiinterleukin 1 antibodies in experimental arthritis. *J Rheumatol* 1992; **19**: 348–356.
20. Schwab JH, Anderle SK, Brown RR, Dalldorf FG, Thompson RC. Pro- and anti-inflammatory roles of interleukin-1 in recurrence of bacterial cell wall-induced arthritis. *Infect Immun* 1991; **59**: 4436–4442.
21. Otterness IG, Seymour PA, Golden HW, Reynolds JA, Daumy GO. The effects of continuous administration of murine interleukin-1a in the rat. *Physiol Behav* 1988; **43**: 787–804.
22. Borer KT. Characteristics of growth-inducing exercise. *Physiol Behav* 1980; **24**: 713–720.
23. Daumy GO, Merenda JM, McColl AS, *et al.* Isolation and characterization of biologically active murine interleukin-1α from expression of a synthetic gene in *Escherichia coli*. *Biochem Biophys Acta* 1989; **998**: 32–42.
24. Daumy GO, Wilder CL, Merenda JM, McColl AS, Geoghegan KF, Otterness IG. Reduction of biological activity of murine recombinant interleukin-1β by selective deamidation at asparagine-149. *FEBS Lett* 1991; **278**: 98–102.
25. Otterness IG, Gans DJ. Nonsteroidal anti-inflammatory drugs: analysis for the relationship between laboratory animal and clinical doses, including species scaling. *J Pharm Sci* 1988; **77**: 790–795.
26. van de Loo FAJ, Arntz OJ, van den Berg WB, Otterness IG. Proteoglycan loss and subsequent replenishment in articular cartilage after a mild arthritic insult by IL-1 in mice: impaired proteoglycan turnover in the recovery phase. *Agents Actions* 1994 (in press).
27. Stimpson SA, Dalldorf FG, Otterness IG, Schwab JH. Exacerbation of arthritis by IL-1 in rat joints previously injured by peptidoglycan–polysaccharide. *J Immunol* 1988; **140**: 2964–2469.
28. Banks WA, Ortiz L, Plotkin SR, Kastin AJ. Human interleukin (IL) 1α, murine IL-1α and murine IL-1β are transported from blood to brain in the mouse by a shared saturable mechanism. *J Pharm Exp Ther* 1992; **259**: 988–996.
29. Newton RC, Uhl J, Covington M, Black O. The distribution and clearance of radiolabeled human interleukin-1β in mice. *Lymphokine Res* 1988; **7**: 207–215.
30. Reimers J, Wogensen LD, Welinder B, *et al.* The pharmacokinetics, distribution and degradation of human recombinant IL-1 beta in normal rats. *Scand J Immunol* 1991; **34**: 597–610.
31. Chizzonite R, Truitt T, Kilian PL, *et al.* Two high affinity interleukin 1 receptors represent separate gene products. *Proc Natl Acad Sci USA* 1989; **86**: 8029–8033.
32. Peretti M, Solito E, Parente L. Evidence that endogenous interleukin-1 is involved in leukocyte migration in acute experimental inflammation in rats and mice. *Agents Actions* 1992; **35**: 70–78.
33. Otterness IG, Bliven JL, Carreras I, Sipe JD. *In vivo* roles of IL-1α and IL-1β. *Cytokine* 1991; **3**: 513.
34. Long NC, Otterness IG, Kunkel SL, Vander AJ, Kluger MJ. Roles of interleukin 1b and tumor necrosis factor in lipopolysaccharide fever in rats. *Am J Physiol* 1990; **28**: R724–R728.
35. Sapadaro F, Dunn AJ. Intracerebroventricular administration of interleukin-1 to mice alters investigation of stimuli in a novel environment. *Brain Behav Immunol* 1990; **4**: 308–322.
36. Dunn AJ, Antoon M, Chapman Y. Reduction of exploratory behavior by intraperitoneal injection of interleukin-1 involves brain corticotropin-releasing factor. *Brain Res Bull* 1991; **26**: 539–542.
37. Hukkanen M, Grönblad M, Rees R, *et al.* Regional distribution of mast cells and peptide containing nerves in normal and adjuvant arthritic rat synovium. *J Rheumatol* 1991; **18**: 177–183.
38. Mapp PI, Kidd BL, Gibson SJ, *et al.* Substance P-, calcitonin gene-related peptide- and C-flanking peptide of neuropeptide Y-immunoreactive fibers are present in normal synovium but depleted in patients with rheumatoid arthritis. *Neuroscience* 1991; **37**: 143–153.

ACKNOWLEDGEMENT. The authors thank Deborah Scampoli for cutting and preparing the tissue sections.

**Received 26 January 1994;**
**accepted in revised form 28 February 1994**
